# Dentistry Qualifications

**Published:** 1906-11-03

**Authors:** 


### Dentistry Qualifications.

The General Medical Council require dental students to pass a preliminary examination similar to that required for medical students and to register as a dental student. From the date of registration the student must be engaged for four years in professional studies, including two years' instruction in mechanical dentistry from a registered practitioner or in a dental hospital. During his curriculum the student must attend courses similar to those of medical students in chemistry, anatomy, physiology, surgery, medicine, and clinical work, and as special subjects dental anatomy and histology, dental surgery and pathology, dental mechanics, and attend two years at a dental hospital operating and working according to the regulations. The following bodies grant dental diplomas:—

1. Royal College of Surgeons—L.D.S.(Eng.). There are three examinations: (*a*) Preliminary Science Examination, fee £3 3s.; (*b*) First Professional, fee £2 2s.; (*c*) Second Professional, fee, Part I. £2 2s., Part II. £3 3s. A further £10 10s. is paid for the diploma. For further information write to F. G. Hallett, Examination Hall, Victoria Embankment, London, W.C.

2. University of Birmingham grants degrees B.D.S., M.D.S., or L.D.S. Fees for L.D.S. and B.D.S., £146 19s., exclusive of books and instruments.

3. University of Leeds grants diploma L.D.S., on the General Medical Council's conditions.

4. Liverpool University grants degrees similar to the University of Birmingham.

5. University of Manchester also grants dental degrees and diplomas.

### Scotland.

6. The Royal College of Surgeons, Edinburgh: For L.D.S.(Edin.) two examinations are taken during the collegiate curriculum—namely, First in anatomy, chemistry, physics, and physiology; Second dental surgery, medicine, and therapeutics. Fees: £15 15s. Address: Mr. James Robertson, 54 George Square, Edinburgh.

7. The Faculty of Physicians and Surgeons, Glasgow. The examinations and fees as in Edinburgh. Address: Dr. Alexander Duncan, B.A., 242 St. Vincent Street, Glasgow.

### Ireland.

8. Royal College of Surgeons (Ireland). The L.D.S.(Ire.) is granted to candidates who pass two professional examinations in the subjects and under conditions prescribed by the General Medical Council. Fees: £21. Address: The Registrar, Royal College of Surgeons, Stephens Green, Dublin.

9. The University of Dublin grants both a degree (M.Dent.Sc.) and a licence (L.Dent.Sc.) in dental surgery. For the degree the candidates must have the B.A., Dublin, and have their names on the medical or dental register for five years. The subjects pathology, bacteriology, and human and comparative dental anatomy are not required for the licence, and the conditions of entry are not the same as for the degree.

### The Cost of Qualification.

After passing the preliminary examination the student should be articled to a registered dentist for two years and enter his name on the students' register.

### INSTITUTIONS FOR DENTAL INSTRUCTION.

### London.

*Charing Cross Hospital Dental Department*, Chandos Street, London, W.C.—Fees for the two years' curriculum required by dental students are 55 guineas in one sum, or 61 guineas in two instalments. Dean, Dr. Christopher Addison.

*Guy's Hospital Dental Department and School*, London Bridge, S.E.—Fees for L.D.S.(Eng.), £110 (or £115 10s. in two instalments). Dean, Dr. H. L. Eason.

*National Dental Hospital and College*, Great Portland Street, W.—Fee for the two years' dental hospital practice required by the curriculum, £21. Fee for full course of dental lectures required by the curriculum, £21. Total fee for the special dental lectures and hospital practice required by the curriculum, £42, in two annual instalments of £21, or one payment of £40. The fees for the necessary general hospital practice and lectures vary from about 55 to 65 guineas, particulars of which are to be found in the calendars of the several schools. The minimum cost of the required education for the L.D.S.Eng. as arranged at the National Dental Hospital is as follows: General hospital (say), £57; dental hospital, £40; books, instruments, etc. (say), £30 19s.; diploma, £21; bacteriological laboratory fee, £1 1s.; total, £150. The above does not include the premium which may be paid for the necessary two years' pupilage in dental mechanics. If this be taken in the mechanical laboratory attached to the hospital, the fee is £50 per annum. N.B.—In consequence of the new regulations of the Royal College of Surgeons, the mechanical pupilage has been reduced from three years to two years, which has caused the composition fee to be reduced to the sum stated opposite. A composition fee has been arranged covering both the *two years' mechanical pupilage* and the two years' hospital practice and lectures required by the curriculum of the Royal College of Surgeons, amounting to £120 in one payment on entrance, or 120 guineas in three instalments of £63, £31 10s., and £31 10s., payable at commencement of first three years respectively. Dean, Mr. Sidney Spokes.

*Royal Dental Hospital of London*, Leicester pital practice and lectures, £53 3s. Single courses Square, W.C.—Fees for instruction in mechanical dentistry and the two years' hospital practice and lectures, for L.D.S., £150; for the two years' hosmay be taken. Further particulars can be obtained of the Dean.

*St. Thomas's Hospital Dental Department.*—The arrangements and fees are similar to those at Guy's and Westminster.

*Westminster Hospital Dental Department.*—Fees: One payment of £52 10s., or two payments of £27 10s. each.

PROVINCIAL.

*Birmingham.*—University Dental Department and Dental Hospital.

*Bristol.*—Medical School, Royal Infirmary, and General Hospital.

*Dublin.*—School of Dentistry and Dental Hospital of Ireland, Lincoln Place.

*Edinburgh.*—Dental Hospital and School, 31 Chambers Street. Dean, Mr. W. Guy, F.R.C.S., 11 Wemyss Place.

*Exeter.*—Dental Hospital. Hon. Secretary, Mr. Henry Yeo, 8 Bedford Circus, Exeter.

*Glasgow.*—Dental Hospital and School, 5 St. Vincent Street.

*Leeds.*—University School of Dentistry.

*Liverpool.*—University Dental Department and Dental Hospital.

*Manchester.*—University Dental Department and Victoria Dental Hospital.

*Newcastle-on-Tyne.*—Royal Infirmary and Dental Hospital.

*Sheffield.*—The University Dental Department.

The following is the syllabus of one month's course of dentistry suitable for the Colonial medical service provided by the National Dental Hospital and College, Great Portland Street, W.

The fee is £5 5s. The course includes the following subjects:—Anatomy of the tooth, including its relations to the jaw; general pathology of dental caries; diagnosis of odontalgia and neuralgia; differential diagnosis; instruments.

Materials for treating teeth:—(*a*) Drugs and dressings; (*b*) for filling cavities.

Methods of treatment:—(*a*) Exclusion of saliva; (*b*) preparation and filling of simple cavities; (*c*) preparation and filling of compound cavities; (*d*) in exposure of pulp. Stomatitis:—(*a*) Local; (*b*) general. Qualified medical men are also admitted for a course of one month in the anæsthetic department, for a fee of £3 3s., applications for which have to be made to the medical committee.

Applications to be addressed to Sidney Spokes, Esq., Dean of the College.

